# The PI3K signaling pathway as a pharmacological target in Autism related disorders and Schizophrenia

**DOI:** 10.1186/s40591-016-0047-9

**Published:** 2016-02-11

**Authors:** Lilian Enriquez-Barreto, Miguel Morales

**Affiliations:** Institut de Neurociències, Departament de Bioquímica i Biologia Molecular, Universitat Autònoma de Barcelona, Barcelona, Spain

## Abstract

This review is focused in PI3K’s involvement in two widespread mental disorders: Autism and Schizophrenia. A large body of evidence points to synaptic dysfunction as a cause of these diseases, either during the initial phases of brain synaptic circuit’s development or later modulating synaptic function and plasticity. Autism related disorders and Schizophrenia are complex genetic conditions in which the identification of gene markers has proved difficult, although the existence of single-gene mutations with a high prevalence in both diseases offers insight into the role of the PI3K signaling pathway. In the brain, components of the PI3K pathway regulate synaptic formation and plasticity; thus, disruption of this pathway leads to synapse dysfunction and pathological behaviors. Here, we recapitulate recent evidences that demonstrate the imbalance of several PI3K elements as leading causes of Autism and Schizophrenia, together with the plausible new pharmacological paths targeting this signaling pathway.

**PI3K signaling pathway and neurological diseases**

## Background

The phosphoinositide 3-kinase (PI3K) signaling is one of the pathways controlling cell survival, proliferation and apoptosis. Mutations on this pathway are often found in cancer cells, favoring tumor cell survival and spreading [1]. When appearing in neuronal tissue, similar mutations result in a different phenotype affecting neuronal morphology and synaptic transmission and, in some cases, severe learning and behavioral imbalances [2]. For instance, mutations in PTEN, neurofibromatosis (NF1) or in the tuberous sclerosis complex, cause an overactivation of the PI3K/Akt/mTOR pathway, leading to autism-related behavior, tuberous sclerosis and macrocephaly [3, 4]. Furthermore, Fragile X syndrome (FXS), a common inherited form of mental retardation and the leading cause of autism, has been associated with overactivation of the PI3K-mTOR pathway [5]. The opposite is also true; low levels of PI3K/Akt/mTOR activity are linked with Rett syndrome (RTT), a rare case of autism-associated disease [6].

Altered PI3K signaling pathway has also been associated with schizophrenia [7–9]. Genetic susceptibility factors such as Neuregulin-1 (NRG1) and its receptor ErbB4, Disrupted-in-Schizophrenia-1 (DISC1) and Dysbindin-1, regulate PI3K/Akt signaling [9, 10]. The NRG1 receptor ERBB4 exists in different isoforms depending on its extracellular juxtamembrane domain or C-terminal cytoplasmic tail (CYT) [11]. The CYT-1 isoform of Erb4 contains a PI3K binding site that activates PI3K signaling [8, 12]. Hence, deregulation of the NRG1/Erb4/PI3K pathway leads to increased levels of CYT-1 and of the catalytic PI3K subunit p110δ (PIK3CD) [8]. NRG1 also regulates DISC1 expression, and it is required for DISC1 maintenance during cortical development. The mechanism is mediated via ErbB2/3 receptors and PI3K/Akt signaling [13]. A paralog of NRG1, Neuregulin-3 (NRG3) is another risk factor associated with schizophrenia [14, 15]. Interestingly, recent findings have revealed that the molecular machinery underlying NRG3 overexposure involves Akt signaling [15].

AKT1 is considered to be a potentially susceptible gene for the development of schizophrenia. Accordingly, impairment of AKT1/GSK3β signaling in this disorder has been clearly demonstrated [16–18]. The levels of AKT1 are reduced in the prefrontal cortex and the hippocampus of postmortem brains, as well as in the peripheral lymphocytes of individuals with schizophrenia [18]. A disturbance in growth factors also seems to contribute to an aberrant PI3K/Akt pathway and the pathogenesis of the disease [9].

Synaptic dysfunction is a common hallmark in ASD and Schizophrenia. Interestingly, during the last decade it has been increasingly highlighted the role of PI3K signaling in the modulation of synaptic plasticity [19–22]. Thus, a relationship between altered PI3K pathway and synapse pathology may be established.

In this article, we give an overview of some well-characterized mutations in several elements of the PI3K molecular cascade and their importance in autism and schizophrenia.

### PI3K-Akt signaling pathway

**Fig. 1:**
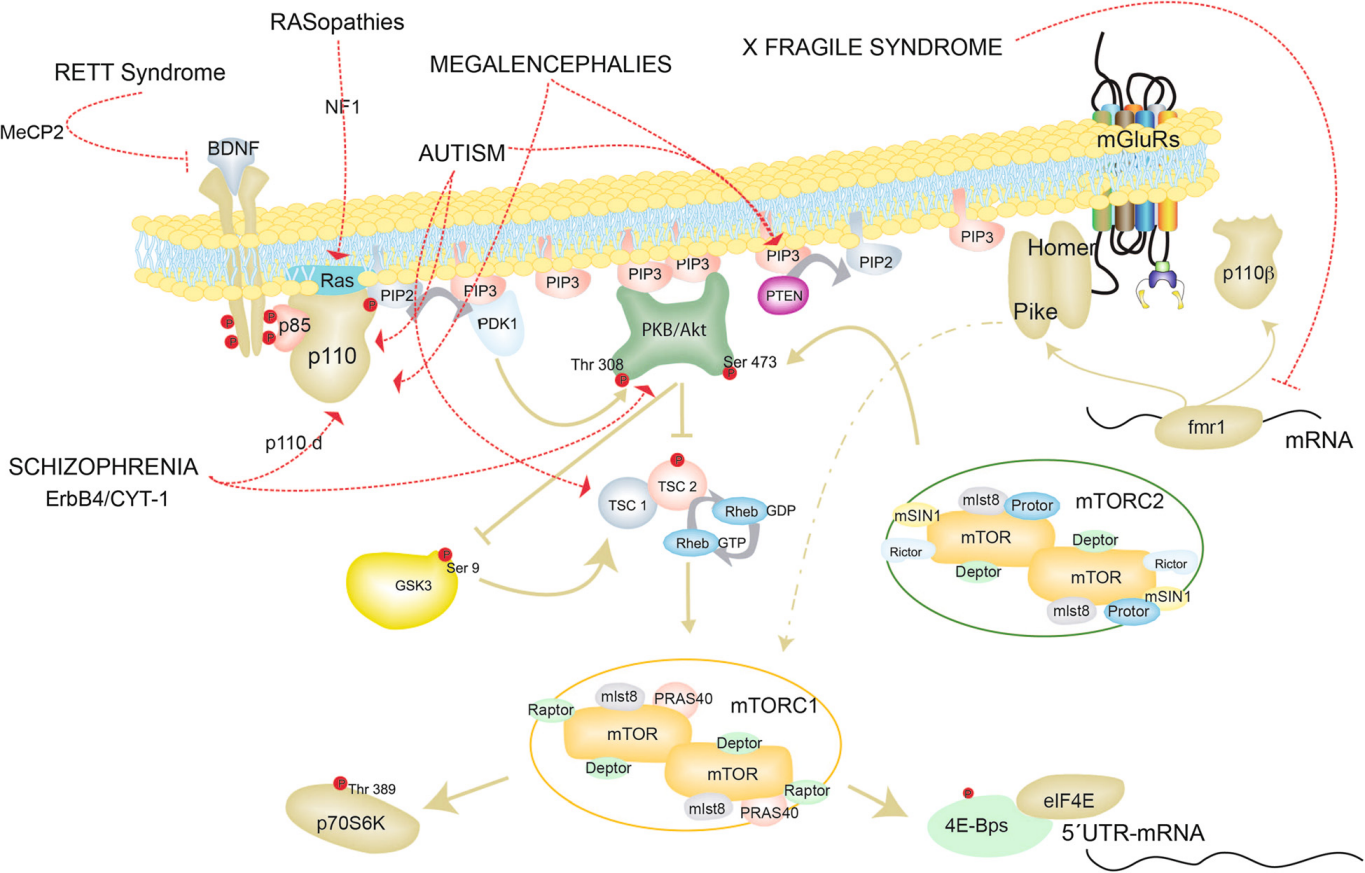
PI3K signaling and common mutations related with ASD and schizophrenia. Class I PI3K is activated by growth factors through a tyrosin kinase receptor. The PI3K activation results in the conversion of PtdIns [4, 5]P_2_ (PIP2) to PtdIns [3–5]P_3_ (PIP3), a process that is reversed by the action of PTEN. PIP3 serves as docking for Akt and PDK1. Akt, indirectly, stimulates mTORC1 resulting in an augment of protein synthesis by phosphorylation of the ribosomal kinase p70S6K or 4E-BPs. Phosphorilation of the former releases its binding to eIF4E and enhances translation. The mTOR kinase is encoded by a single gene in mammals, but it is the active enzyme in two multi-protein complexes called mTORC1 and 2. mTORC1 is defined by the Raptor subunit (regulatory-associated protein of mTOR) while mTORC2 by the Rictor (rapamycin-insensitive companion of mTOR). mTORC2 is also activated by growth factors through a not well-defined PI3K-dependent mechanism. mTORC2 contributes to the full activation of Akt by phosphorylation on serine 473 (for a review see [156]). An important kinase downstream of PI3K is GSK3, which is inhibited by direct Akt phosphorylation. Among other functions GSK3 regulates TSC activity and indirectly protein translation. Several signaling pathways dramatically alter PI3K activity. Downstream mGLUR5, fmr1 regulates transcriptional levels of PIKE or the catalytic PI3K subunit p110β. Mutations in the phosphatase PTEN enhance mTOR dependent translation. Mutations on NF1 enhance Ras and p110 catalytic activity. Lack of MecP2 expression reduces BDNF levels that in turn contributes to a general deficit of PI3K signaling. Mutations in the PI3K catalytic subunit p110δ or Akt3 isoform are associated with schizophrenia. Elevated levels of CYT-1 expression, one of the isoforms of the ErbB4 receptor, raised expression of the p110δ subunit and are also connected with schizophrenia

PI3K, first discovered by Lewis Cantley and colleagues [23, 24], constitutes a family of intracellular lipid kinases that phosphorylate the 3-OH of the inositol ring of phosphatidylinositols (PtdIns) at the plasma membrane lipids. Three classes of PI3Ks can be found in mammals, and all three are expressed in the mammalian central nervous system. Class I comprises p110α (PIK3CA), p110β (PIK3CB), p110γ (PIK3CG) and p110δ (PIK3CD) and it is further divided into IA and IB. IA includes the p110α, p110β and p110δ that bind the p85 regulatory subunit (PI3KR1), while Class IB consists of p110γ [25]. Class II includes three different types, C2α, C2β and C2γ. Finally, there is PI3K-C3, the only member of the ubiquitously expressed Class III, also named vacuolar protein sorting 34 (Vps34) [26, 27]. Class I, perhaps the best-studied and characterized, is a heterodimer composed of one regulatory (p85) and one catalytic subunit (p110). Class I activation might rely on a tyrosine kinase receptor (IGF-1, EGF) or on a G-protein coupled receptor, such as the metabotropic glutamate receptors. However, full p110 catalytic activity requires Ras activation through a Ras-interacting domain present in the p110 sequence [28, 29]. Upon activation, the regulatory subunit interacts with the catalytic subunit through a Src-homology domain 2 (SH2), promoting a conformational change that activates the catalytic subunit, inducing a local increase in PtdIns [3–5]P_3_ at the membrane level, that can subsequently function as an attractor of proteins with a pleckstrin domain, such are Akt (also known as PKB) or PDK1 (Phosphoinositide-dependent kinase-1) (Fig. 1).

Akt is the major downstream effector of PI3K, upon membrane docking and in order to be functional, Akt needs to be phosphorylated by two kinases: PDK1 and mTORC2. Downstream Akt lies the TSC1/TSC2 complex (Tuberous Sclerosis Complex1/2) that constitutively represses mTORC1 activity. TSC1 stabilizes TSC2. TSC2 has a GTPase-activated protein (GAP) domain that stimulates the GTPase activity of the small GTPase Rheb (Ras Homolog Enriched in Brain). Akt activation induces TSC2 phosphorylation and consequently inactivation of the complex due to the disruption of its GTPase function. The resulting GTP-bound Rheb promotes mTORC1 activity (mammalian target of rapamycin 1). Thus, direct phosphorylation of TSC2 by Akt inhibits its functions, and therefore mTORC1 activation (Fig. 1). Mutations in the tumor suppressor genes encoding TSC1 (Hamartin) and TSC2 (Tuberin) lead to a multisystemic tumor syndrome called tuberous sclerosis, which is characterized by neoplastic lesions (i.e., hamartomas) [30].

PI3K actions are antagonized by PTEN (phosphatase and tensin homolog deleted on chromosome 10). PTEN, which was originally cloned as a tumor suppressor protein, dephosphorylates PtdIns(3–5)P_3_ to generate PtdIns(4, 5)P_2_ [31]. PTEN is one of the most frequently mutated proteins found in tumor cells. Null mice for PTEN die during embryogenesis. Interestingly, germline PTEN mutans cause autosomal dominant hamartomas, mental retardation, and in some cases, macrocephaly [32, 33].

### Autism and related disorders as a synaptic dysfunction

Autism comprises a group of disorders that are collectively termed as autism spectrum disorders (ASD). These disorders share similar clinical features including impaired social behavior and language skills, limited range of interests and the exhibition of ritualistic and repetitive behaviors [34]. Recent data indicate that the prevalence of ASD is close to 1.1 % [35]. Cognitive impairment and mental retardation are common attributes of autism (70 %), accompanied by minor and inconstant abnormalities in brain morphology. However, it should be noted that a conspicuous 10 % of ASD patients suffer from macrocephaly [36, 37]. Intriguingly, 10 % of autistic patients show an extraordinary talent, and are often referred to as *idiot savants* [38].

A large body of evidence points to synaptic dysfunction as a cause of the disease [39]. First, most of the behavioral and cognitive disorders become evident prior to 3 years of age, a period characterized by intense synaptic remodeling in the human brain [40]. Second, several ASD-related mutations have been identified in synaptic proteins including Shank3, Homer or PSD95 scaffolds [41]. Third, mutations in the synaptic adhesion molecules neuroligins (NLGS), which control the balance between excitatory and inhibitory neurotransmission in mammalian brains, are associated with ASD [42, 43]. NLGS mutations represent less than 1 % of genetic ASD; however mutations of NLGS receptors, such as NRXN1 (Neurexin-1-alpha gene) and CNTNAP2 (Contactin-associated protein-like 2) have been identified in ASD patients [44–46]. Finally, a high percentage of ASD patients (10–30 %) also suffer from epilepsy [47].

Early genetic studies employing twins suggested that ASD has a genetic origin [48]. More recent studies have estimated that the inheritance of ASD is close to a 50 % [49], although due to the polygenic origin of the disease, the genetic causes have been elusive [50]. Only 8–15 % of all cases of ASD are associated with a mutation located in a single gene, and remarkably, more than half of these mutations are directly or indirectly related to the PI3K/Akt/mTOR pathway. Indirectly supporting the imbalance of PI3K and its relation with autism, some groups have reported a reduction of IGF-1 levels in the cerebrospinal fluid of autistic children [51] and low levels of phospho-Akt in the brain of autistic subjects [52].

### PI3K signaling pathway-associated mutations in ASD

#### *P110*-Akt

As previously mentioned, macrocephaly is a recurrent theme in ASD patients. ASD children born with normal brain size, but during the first six years of life, the circumference of the head undergoes abnormal growth. Although the nature of brain overgrowth is not well understood, the presence of too many neurons, dendrites, spines or even glial cells could explain this clinical feature. Somatic mutations of the catalytic subunit p110α induce megalencephalies and hemimegalencephalies (HME) as well as brain malformations characterized by an overgrowth of either or both of the cerebral hemispheres (HME; 18 % in a cohort of 33 affected children [53]). HME, in addition, are often associated with developmental delays and epilepsy [54, 55]. Another element of the PI3K pathway found in somatic mutations of HME patients is the Akt3 brain kinase isoform. Activating mutations or genomic duplications encoding Akt3 were found in three of eight brain resections from patients with HME [54, 56]. Accordingly, the PDK1-K465E knock-in mice, that express a PDK1 mutation with an impaired membrane phosphoinositides interaction, shows low levels of Akt activation and a reduced brain size [57].

#### PTEN

Loss of function in the negative regulator PTEN has been linked to macrocephaly and ASD. Mutations in PTEN gene have been found in 5 % of ASD patients with macrocephaly [33] and recent studies focused on pediatric patients with ASD found a mutation prevalence rate of 7–8.3 % [58, 59]. Additionally, subjects with developmental delay/mental retardation have a higher prevalence (12.2 %) of PTEN mutations [58]. Work in animal models has revealed that PTEN null mutant mice exhibit embryonic lethality, while the heterozygotes are highly susceptible to tumors [60]. Mammalian primary neuron cultures and Drosophila null mutants of PTEN show extensive dendritic arborization, which highlights the role of the PI3K/AKT/mTOR pathway in the regulation of neuronal growth [61, 62]. Accordingly with the relationship between neuronal overgrowth and ASD, brain-specific conditional PTEN null mice develop dendritic hypertrophy, increased spine density and progressive macrocephaly confined to the affected brain areas. Interestedly, PTEN mutant mice display abnormal social interactions that resemble autism-like behaviors [63, 64]. The abnormal morphology was associated with Akt hyperactivity and overactivation of mTORC1 and its downstream element p70S6K [64]. Chronic treatment with rapamycin (mTORC1 inhibitor) reverted the PTEN-mutant phenotype, suggesting that inhibition of mTORC1 could be a pharmacological target in the treatment of ASD. Interestingly, rapamycin treatment ameliorates the social deficits in the inbred mouse strain BTBR, a non-syndromic model of ASD [65].

#### Tuberous sclerosis and TSC1/TSC2 complex

Tuberous sclerosis complex (TSC) is an autosomal dominant disorder caused by mutations in Hamartin or Tuberin genes [66]. The estimated prevalence of TSC is 6.8–12 per 100 000 persons, with no sex or ethnic differences. The estimated incidence of tuberous sclerosis at birth is one in 5800 newborns [67]. TSC is a multisystem syndrome classically associated with the occurrence of cortical brain dysplasias, called tubers. The effects of these mutations on the brain are associated with high susceptibility to ASD and macrocephaly. Between 25 % and 61 % of individuals affected by TSC meet the diagnostic criteria for autism [68], and 50 % of those with TSC have some type of learning disability [69]. Conversely, mutations in TSC1/2 appear in 1–4 % of all cases of autism [3]. Similar to PTEN mutations, inhibitory mutations in any of the TSC elements of the complex would result in an imbalance in mTORC1 activity, increasing protein translation and modifying neuronal morphology [30, 70]. Mutant flies from the *gigas* gene, a fly orthologue of TSC2, have changes on cell size. Interestingly, *gigas* neurons increase two-fold the number of synapses established with their target neurons [71]. In the other hand, mice with heterozygous mutations in TSC2 show cognitive deficits similar to human TSC patients. In this mouse model, treatment with rapamycin compensates for deficits in context discrimination and spatial memory [72].

### PI3K signaling in Neurofibromatosis type 1 and RASopathies

Ras/mitogen activated-protein kinase (MAPK) and PI3K/Akt pathways are two signaling cascades with a high degree of cross-regulation among their elements [73]. Remarkably, both pathways have been reported to coordinately mediate neuronal survival and axonal growth [74] and to independently or synergistically modulate dendritic arbor and spine morphogenesis [75]. Furthermore, Ras/MAPK and PI3K/Akt pathways are also required for proper neuronal migration during cortical development [76]. Mutations in the genes that encode components of the RAS/ MAPK pathway lead to a special class of disorders commonly referred as RASopathies [77]. This family includes, among others, Noonan syndrome, Costello syndrome or Neurofibromatosis type 1 (NF1). The latter is a familial cancer syndrome caused by an autosomal dominant mutation of NF1 gene, affecting 1/3000-5000 individuals worldwide. NF1 patients suffer from Schwann cells tumor cell affecting peripheral nervous system (neurofibromas) or astrocytomas [78, 79]. In NF1, a mutation disrupts neurofibromin, a GTPase that negatively regulates p21Ras, resulting in an enhancement of Ras activity and upregulation of PI3K [79, 80]. As mentioned, Ras binds directly to p110 catalytic subunit, further modulating its activity [81, 82]. NF1 children are often afflicted with cognitive and social deficits that resemble ASD [83]. A recent study estimates that in the U.K. a total of 29.5 % of NF1 patients were diagnosed with severe ASD whereas 27.7 % with a moderate ASD [84]. Linking NF1 to the process of synaptic regulation, 6.5 % of NF1 patients had documented epilepsy [85]. Interestingly, mice that are heterozygous for the NF1 gene have deficits in visual attention, spatial learning and hippocampal long-term potentiation (LTP). Treatment with lovastatin, a farnesyl transferase inhibitor that blocks Ras membrane attachment, reverses mutation effects [86].

### Deregulation of PI3K signaling in the Fragile X syndrome

FXS syndrome provides evidence that mutations of distant regulatory elements of the PI3K pathway also cause ASD. FXS is an X-linked form of mental retardation that occurs by the loss of function of the X-fragile mental retardation protein (fmr1 [87]). The exact number of people who have FXS is unknown, but it has been estimated that human prevalence is about 1 in 4000 for males and 1 in 6000 for females [88]. FXS is the most common form of inheritable intellectual disability and a genetic leading cause of autism. Indeed, around 15–30 % of FXS patients develop ASD and conversely a 5 % of diagnosed autistic children are found to have FXS. At the molecular level, fmr1 is a translation regulatory element that binds to hundreds of specific mRNAs, repressing their translation (more than 400 have been identified [89]). In humans, FXS results from the expansion of a CGG repeat sequence in the 5′ untranslated region of fmr1, which induces a functional silencing of the gene (5–20 in normal person and more than 200 in affected [90]). The lack of fmr1 causes a broad deregulation of translation, as evidenced by a substantial 20 % increase in the rate of brain protein translation in the FMR1 knockout mice (KO) [91, 92]. Neuroimaging studies of human patients have revealed some morphological brain anomalies, including increased brain size, larger amygdala and hippocampus, as well as ventricular abnormalities, among others [93, 94].

Two prominent features of FXS found in both humans and mouse models are the presence of aberrant dendritic spines and an exaggerated form of Long Term Depression (LTD [95]). The so called “mGluR theory of FXS” postulates that excessive protein synthesis downstream from gp1 mGluRs (group one of glutamate metabotropic receptors) underlies most of the FXS symptoms [96]. Supporting this hypothesis, both genetic and pharmacological targeting of mGluR alleviates FXS manifestations [97, 98]. Therefore, in rodent models, allosteric antagonists of mGluR5 (such as MPEP or AFQ056) have demonstrated a degree of efficiency in both reverting LTD, aberrant spines and ameliorating some of the behavioral defects [98, 99].

The altered elements downstream of mGLuRs are still a matter of study, but numerous components of the PI3K/Akt/mTOR pathway appear to be clearly upregulated in the absence of functional fmr1 expression. Experimental evidence confirms this point: FMR1 KO mice show elevated levels of phospho-Akt, phospho-mTOR and phospho-p70S6, as well as excessive mTOR cap-dependent translation and deregulated LTD [5, 96, 100–102]. Even human postmortem tissue shows altered mTOR signaling evidenced by enhanced phosphorylation of Akt levels (upstream mTOR regulator) and S6K1 (downstream mTOR substrate) [103]. Conversely, inhibition of protein synthesis with mTORC1 inhibitors such rapamycin or temsirolimus, reduces basal protein synthesis and ameliorates cognitive deficit, measured by the object recognition test, as well as susceptibility to audiogenic seizures [104].

Levels of PIKE (PI3K enhancer), an upstream activator of mTOR and an identified mRNA target of FMR1, are elevated in FMR1 KO mice. PIKE should link mGluR activity with PI3K signaling; nonetheless, it has been suggested that this upregulation underlies the high content of mTOR-dependent translation found in FXS synapses [101]. PIKE is not the only element of the PI3K pathway upregulated in FXS neurons; p110β, the catalytic isoform of PI3K Class I is augmented in mouse neurons deficient in Fmr1 and in lymphoblastoid cells from FXS patients, causing excessive protein synthesis [5, 105]. Specific inhibitors of the p110β subunits, such as TGX221, restore normal protein synthesis in cortical synaptosomes of FMR1 KO mice and reduce Akt activity in the lymphocytes of human patients, without affecting wild type cells [105]. Alternatively, altered PI3K/Akt/mTOR may not be the cause, rather the consequence of the lack of fmr1 activity, nevertheless, elements downstream mGluR activation appears to be promising pharmacological targets in FXS.

### A particular case of PI3K signaling imbalance: Rett syndrome

Rett syndrome (RTT) is a monogenic X-linked disease with a prevalence of 0.4 per 10000 females [106]. RTT shares a high co-morbidity with ASD, it develops during the first 6–18 months of life, and its clinical features include progressive mental decline, autism and stereotyped movements. Notably and different from ASD, patients suffer from a severe deregulation of the motor and sensory system and an important disturbance of the autonomous nervous systems, producing an irregular heart rate and breathing patterns that will eventually result in the death of the patient. At the histological level, RTT is characterized by a series of features that include a reduction of cortical thickness, decreased volume of neurons, fewer dendritic branches and poor development of spines.

The syndrome is mainly caused (90 %) by a mutation in the methyl-CpG-binding protein2 gene (MeCP2 [107]). MeCp2 is ubiquitously expressed, although the brain displays the highest levels of expression [108], with similar amounts of expression in both neurons and astrocytes [109].

MeCp2 is a methylation binding protein that selectively binds to specific DNA regions, inhibiting transcription [107, 110]. It has thus been hypothesized that MeCp2 is a chromatin-silencing regulator [111]. Recently, a more complex picture has emerged, because MeCp2 can act as a transcriptional activator through the cAMP-Response Element Binding protein (CREB), and as a RNA-splicing modulator, regulating the expression of miR-132/212, both of which are known to affect visual cortical plasticity and ocular dominance [112]. MeCP2 indirectly modifies the activation level of PI3K by regulating the transcription of IGF-1 and BDNF. In this way, MeCp2 participates in a complex and not well-understood regulatory cycle that involving miR-132/212 and CREB, modulates IGF1 and BDNF expression, which in turn activates PI3K [113, 114].

In humans, MeCp2 mutant embryonic stem cells display low levels of p-Akt and p-S6, and overall reduced transcription and translation levels [115]. Similarly, the brains of MeCp2 knockout mice show reduced levels of p-Akt and two markers of protein synthesis, such as Rack1 and eIF6 [116, 117]. BDNF expression is reduced in knockout MeCp2 mice [118, 119] and in human patients [120]. Conversely, restoring BDNF activity improves physiological functions and survival. Thus, in RTT mice models, treatments with promoters of BDNF synthesis, such as sphingosine or a druggable intercellular loop of the TrkB receptor, improve locomotor activity and survival in MepC2 deficient mice [121, 122]. The pharmacological target of IGF-1 also ameliorates Rett symptoms, while treatment of MeCp2-deficient mice with an active fragment of IGF-1 increases their life span, improves locomotor functions, ameliorates breathing patterns and normalizes the heart rate [123]. Similar results were obtained by injecting recombinant human IGF-1 in a mouse model, which improved respiratory patterns, reduced anxiety and increased exploratory behavior. Augmented levels of phosphorylated Akt were found in cortical homogenates of MeCp2-deficient mice after treatment, an effect that parallels the augmented PSD95 synaptic amounts observed in these animals [124]. More recently, findings from the same group have demonstrated the effects of mecarsermin (a druggable recombinant human IGF-1) in human patients. The results of a short-term treatment consisting of a daily dose administered for 4 weeks showed that the use of IGF-1 is safe and it ameliorates irregular breathing and anxiety, although it did not improve intellectual disabilities. Nonetheless, this result clearly paves the way for the use of the PI3K activators in the treatment of Rett syndrome [125].

### Impaired PI3K signaling in schizophrenia

Schizophrenia is a severe psychiatric disorder affecting approximately 1 % of the population worldwide [126]. It is characterized by symptoms such as hallucinations, delusions, apathy and social withdrawal, reduced motivation, disorganized communication and cognitive impairments [127–129]. Clinical symptoms of schizophrenia are usually manifested during adolescence and early adulthood [126], although at present, seminal evidence supports the idea that schizophrenia has a neurodevelopmental origin [130].

#### PI3K

Dysfunction in the PI3K signaling pathway has been connected with the pathogenesis of schizophrenia [7, 8, 10]. Accordingly, family-based association studies of schizophrenia have revealed PI3K class 3 (PIK3C3) genetic contributions to the pathogenesis of the disorder [131]. Genetic analysis of the promoter region of PIK3C3 in patients with schizophrenia pointed to a mutation that probably results in diminished gene transcription [132]. Other studies of gene expression patterns in peripheral blood leucocytes of schizophrenic individuals evidenced a decrease in the expression of the PI3K catalytic subunit-α (P110α, PIK3CA) [133].

Two susceptibility factors for schizophrenia, NRG1 and DISC1, have been demonstrated to be linked by a common pathway involving PI3K/Akt. It is interesting to note that animal models of NRG1 and DISC1 exhibit similar phenotypes, such as impaired prepulse inhibition and working memory deficits. These similarities can be now explained in terms of the ability of NRG1 to mediate DISC1 expression via ErbB2/3 and PI3K/Akt signaling [13]. Furthermore, a risk pathway has been recently reported in schizophrenia that involves NRG1, the ErbB4 receptor isoform CYT-1 and the p110δ catalytic subunit of the IA PI3K class [8]. Thus, in human brains and in lymphoblastoid B-cell lines (LCLs) of subjects with schizophrenia, an ErbB4 risk haplotype (AGG; rs7598440, rs839523, rs707284) was found to be associated with elevated ErbB4 CYT-1 transcription and consequently raised expression of PIK3CD (p110δ). Importantly, despite augmented PIK3CD levels, PI3K signaling is reduced; this surprising result points to a complex deregulation of the NRG1/ERBB4/PI3K pathway, with a negative effect on the PI3K signaling in this disease [8, 134]. Law and colleagues cite similar results regarding elevated levels of PIK3CD, accompanied by dampened PI3K signaling in neuroblastoma tumors [135, 136], an effect suggesting that PIK3CD may act as a tumor suppressor. Quantitative analysis of gene transcripts reveals an increased expression of the catalytic subunit PIK3CD and the regulatory subunit PIK3R3 (p55γ), although data correlation studies indicate that PIK3CD is the subunit genetically implicated in this disorder [8].

Although PIK3CD mRNA expression in the hippocampus and prefrontal cortex of normal individuals is associated with the risk haplotype of ErbB4, this association is not observed in schizophrenic subjects in the hippocampus or the dorsolateral prefrontal cortex, an effect that could be the consequence of antipsychotic drug treatment [8]. Consistent with this hypothesis, haloperidol administration in rats reduced PIK3CD gene expression in the brain [8]. Such findings by Law and colleagues indicate that specific inhibition of the PIK3CD protein (p110δ) may represent a useful approach for the treatment of schizophrenia. Therefore, the effect of small molecule IC87114, a compound that selectively inhibits p110δ catalytic activity, was tested in a model of amphetamine-induced hyperlocomotion in rodents. Its antipsychotic potential was confirmed as a result of a dramatic blockade of the amphetamine-induced hyperlocomotion. Contrary to hypokinesia caused by antipsychotic drugs, the administered dose of IC87114 had no effect on the spontaneous locomotor activity in the absence of amphetamine [8]. The IC87114 compound was also found to modulate Akt activity in mice by significantly increasing Akt phosphorylation levels on Thr308, thus confirming the specific action of IC87114 on the PI3K signaling pathway [8]. The therapeutic potential of IC87114 was also addressed in a model of rat neonatal ventral hippocampal lesion (NVHL), a neurodevelopmental animal model of schizophrenia. These animals exhibit altered prepulse inhibition (PPI) of the acoustic startle response. IC87114 treatment induced a highly significant improvement of PPI in these rats, as compared to sham-operated animals. In general, the effects of IC87114 seem to be specific to the p110δ catalytic subunit and present no side effects [8]. Altogether, the data confirm that p110δ selective inhibition has a promising antipsychotic potential.

Neuregulin 3 (NRG3), a paralog of NRG1, is a molecule of increasing interest, due to its link to psychiatric disorders that includes schizophrenia. Accordingly, elevated NRG3 expression in the prefrontal cortex of schizophrenic subjects has been described [14]. In a set of experiments, an NRG3 EGF peptide consisting of the EGF domain of human NRG3 was administered during a critical neurodevelopmental period in mice. The effect of the treatment produced anxiety-like behavior and reduced sociability in adulthood, thus demonstrating that overexposure to NRG3 in early postnatal life has a negative impact on brain development. The viability of the peripherally injected peptide indicated activation of its ErbB4 receptor and Akt signaling in a way similar to NRG1 [15], confirming the involvement of the PI3K pathway.

#### Akt

Dopamine D2 receptors (D2Rs) have been classically considered as the principal target of antipsychotic drugs [137]. Schizophrenic subjects present high levels of D2Rs in the basal ganglia [137], and the sensitivity of postsynaptic D2Rs is enhanced in this disorder [137, 138]. Antipsychotic drugs can be grouped into two categories, namely typical and atypical. Both groups of drugs are able to modify Akt signaling in schizophrenia [9, 137]. In fact, Akt plays a role in the pathogenesis of the disease. The associations between schizophrenia and AKT1 genetic variants have been well established [16, 139, 140], and decreased levels of Akt1 and substrate phosphorylation have been reported in the brain of individuals suffering from the disorder [16, 17].

Lymphocyte-derived cell lines from schizophrenic subjects exhibit reduced levels of Akt [16]. Analysis of postmortem brains confirmed lower amounts of Akt1 in the frontal cortex and the hippocampus [16]. Phosphorylation of GSK3β at Ser9 in lymphocytes and frontal cortex lysates was also reduced, consistent with a decrease in Akt1 activity. Sequencing of the AKT1 locus in schizophrenic individuals revealed an AKT1 haplotype associated with lower Akt1 protein content [16].

To gain insight into the direct involvement of Akt in schizophrenia, Emamian and colleagues treated Akt1 −/− adult mice with amphetamine, a nonselective dopamine receptor agonist. PPI was then recorded in the mice as a measure of sensory motor gating. Animals deficient in Akt1 exhibited a significant reduction of PPI, thus suggesting impairment of the neuronal circuits that gate the startle reflex. [16] Further analyses were carried out using intraperitoneal administration of haloperidol, a specific D2Rs antagonist, in adult C57Bl/6 mice. Haloperidol usage affected enzymatic Akt activity, and both chronic and acute treatments modulated Akt phosphorylation levels. The acute medication produced an increase in Thr308 phosphorylation; however, chronic administration augmented the phosphorylation levels at Thr308 and Ser473, consequently increasing GSK3β at Ser9 phosphorylation [16]. These observations evidenced that antipsychotic drugs could compensate for the disruption of the Akt/GSK3β pathway in schizophrenia.

Some studies indicate that glucose metabolism is compromised in schizophrenia [141, 142]; hence, detection of the insulin receptor and Akt activity in postmortem brains of schizophrenic patients, in insulin knockdown HEK cells and in a mouse model of insulin resistance, revealed a significant reduction in the number of insulin receptors and in the total and active forms of Akt. The findings confirm that altered insulin receptors activity directly impairs insulin/Akt signaling in this disease [17].

By using Akt1-deficient mice, Lai and colleagues (2006) studied the effect of Akt1 deficiency on prefrontal cortex functioning. Thus, transcriptional profiling examination evidenced changes in the expression of prefrontal cortex genes involved in actin polymerization, synaptic transmission, neuronal development and myelination. Furthermore, changes in dendritic architecture and complexity of prefrontal layer V neurons suggested altered neuronal connectivity. Akt1-mutant mice also displayed abnormal working memory retention [143].

A recent *in vitro* approach has demonstrated that paliperidone, a second-generation antipsychotic, may act as a neuroprotector. Cultured mouse embryonic prefrontal cortical neurons were exposed to the NMDA receptor antagonist MK-801, a compound widely used to model schizophrenia features in animals. MK-801 notably affected cell viability in a dose-dependent manner, but paliperidone effectively prevented neuronal damage. This protective effect implies Akt/GSK3β activity [144].

### Discussion on pharmacological interventions on the PI3K pathway

ASD and schizophrenia, although etiologically different, occur with synaptic dysfunction and disruption of neuronal networks. Both pathologies have a developmental origin that might result in synaptic plasticity deficits and the malfunction of neuronal circuits throughout the subject’s lifespan. Krueger and Bear [97] have established three possible scenarios for the treatment of FXS: optimal, pessimistic and hopeful, grading the success of a future treatment as a function of the timing and irreversibility of the impairments [97]. We believe that a similar scenario could be extended to include ASD and schizophrenia treatments. A key remaining issue is the temporal window in which reverting the disease, or if established, ameliorating the symptoms, is the suitable choice.

Being optimistic in terms of research progress, we would like to contribute to the discussion of a hopeful scenario in which pharmacological treatments can slow or even reverse the evolution of symptoms in these neurological disorders. There are two main reasons for this. First, some of the treatments already tested in mouse models of ASD have been able to ameliorate both cognitive and morphological alterations; moreover, a few clinical tests with humans show promising results (this is true at least for the use of mGluR5 regulators in FXS or PI3K activators for Rett syndrome treatment). On the other hand, most of the cellular mechanisms regulating neuronal plasticity during development are also present later in adolescence and adulthood [145, 146].

The neuropathological features of ASD include altered cellular size and synaptic growth, synaptic plasticity failure, changes in synaptic proteins, dendritic spine dysmorphology and abnormal synaptic homeostasis [3, 147, 148]. Schizophrenia neuropathology comprises ventricular enlargement, neuron and brain atrophy, reduced dendritic arborization and hypoplasticity [7]. It is interesting to consider that the PI3K pathway is involved in neuronal migration and cortical lamination [149], the regulation of dendritic branching [19, 61], synaptogenesis and spinogenesis [19–21, 150], the control of functional synaptic plasticity [151, 152] and long-term potentiation in the hippocampus [153]. Thus, allowing for the spectrum of neurological aspects comprised in the pathological conditions here reviewed, as well as the molecular mechanistic convergence on PI3K, a promising clinical aim could be the selective targeting of PI3K signaling.

Since mutations in several elements of the PI3K pathway have been associated with susceptibility to develop cancer, a large number of clinical trials targeting PI3K elements are currently underway [154]. Several specific inhibitors of the p110 catalytic subunit have already been developed. For instance BYL719 (inhibitor of p110α), GSK2636771 (inhibitor of p110β) or inhibitors of p110δ such TGR 1202 are under clinical evaluation for the treatment of different malignant lymphomas. CAL-101 (also known as idelalisib) has already been approved as treatment for three types of β cell neoplasm [155]. None of these drugs has been tested so far for the treatment of psychiatric or neurodevelopmental disorders. More surprisingly, none of these studies is exploring cognitive parameters.

In addition to the large number of cancer cell trials employing rapamycin, the mTOR inhibitor, two clinical trials are currently focused on the use of this drug to treat TSC and autism in children. The use of lovastatin in individuals with NF1 mutations is yet another example of crossing pathways for future research; a clinical Phase I trial is currently concluding, although this study focuses on safety, not neurophysiologic deficits.

Several clinical studies on treatments for FXS targeting mGluR have already been completed. Regrettably, a recent Phase II trial employing mGluR5 antagonist mavoglurant (AFQ056) has been canceled because the study did not show a significant improvement of abnormal behaviors in adults and adolescents with FXS (source: www.fraxa.org). Another mGluR5 antagonist (acamprosate) is now in clinical trials, studying social impairments associated with FXS. To date, no clinical study has contemplated the use of inhibitors/regulators of PI3K pathway elements for the treatment of ASD.

Probably the most promising field is the treatment of Rett syndrome. This monogenetic disease has a clear and well-identified origin and several vegetative dysfunctions that make it easy to monitor progression during treatment. As previously stated, a clinical Phase I/II study employing mecaserim has been completed [125], and a new study also employing human recombinant IGF-1 is already in the recruiting phase. A second clinical trial (Phase I) is now in its initial phases to study the safety and tolerance of fingolimod in children with Rett syndrome.

Nowadays, no clinical trials targeting PI3K elements to treat Schizophrenia are underway. We hope that the aforementioned clinical efforts should eventually fuel the development of new molecules for the treatment of this disease based on selective targeting of PI3K elements. In summary, the studies discussed here open the door to develop novel therapeutic molecules targeting PI3K-specific subunits or specific elements of the PI3K/Akt pathway.

## Conclusions

- Unbalance of PI3K signaling is associated with ASD and Schizophrenia and it may drive synaptic dysfunction in both disorders
- Pharmacological targeting of PI3K or related elements could be part of new and promising tools for ASD and Schizophrenia treatment
